# Vygotskian Business Ethics: The Influence of Peers on Moral Reasoning in Business Ethics Education

**DOI:** 10.1177/1052562921996019

**Published:** 2021-03-20

**Authors:** David Ohreen, Binod Sundararajan, Valerie Trifts, Scott Comber

**Affiliations:** 1 Mount Royal University, Calgary, Alberta, Canada; 2 Dalhousie University, Halifax, Nova Scotia, Canada

**Keywords:** business ethics education, Vygotskian constructivism, peer discussions, moral development

## Abstract

The Russian developmental psychologist Lev Vygotsky provides important theoretical underpinnings for an alternative to business ethics pedagogy. Although Vygotsky’s constructivist approach has been applied to other disciplines, such as cognitive development, moral development, and network analysis and learning, its application to business ethics education is virtually nonexistent. Vygotsky’s focus on language and peer influence provides a novel approach to ethics education. Although many business ethics instructors already use group discussion in their classes, we provide evidence that will reinforce such techniques as a crucial pedagogical method. This study is an exploratory application of Vygotsky’s developmental theory to business ethics education. Data were gathered in business ethics and management courses, with experimental and control groups, and analyzed using the Defining Issues Test and thematic-coded journal entries. Results indicated that discussions created a zone of proximal development improving the moral reasoning for most students giving them multiple perspectives and providing support to engage in deliberations and peer dialogue when discussing ethical frameworks, ethical scenarios, and ethical decision making.

## Introduction

Global business environments and internationalization of education present challenges to management education because they are largely imbued with Western or Anglo-American values tied to business schools (Hardy & Tolhurst, 2014). As Giacalone and Thompson (2006) state, “Business educators walk a road where ethical signposts are unclear, new scandals lead to new laws, and society’s increased expectations change the parameters for what and how we teach” (p. 266). Studies have shown that ethics education has not systematically improved the moral reasoning of business students and professionals, and therefore, its effectiveness should be deeply questioned (Ohreen, 2013). Business ethics education has limited effect, in part, because it rests on Western rationalistic traditions within normative ethics, business theory, and cognitive psychology. Emphasis is usually placed on students rationally thinking about issues as a way of improving their critical analysis and reasoning skills. Normative approaches to business ethics as part of this rationalistic tradition has dominated business schools as a way of combatting unethical behavior. This normative approach often rests on an ethical theory applied to case studies and then analyzed through the minutia of arguments for and against. Recently, these traditional philosophical approaches have given way to behavior ethics (de los Reyes et al., 2017). Behavioral ethics tends to focus on empirical social scientific research to understand the many influences on it instead of embracing philosophical analysis. Philosophical ethics does not exhaust normative thinking and, therefore, appeals to behavior research (e.g., emotions, intuition) can broaden our understanding of human nature in real-life scenarios. These learnings can then be applied through the lens of moral prescriptions. What is needed is an integrated approach of both philosophical and behavioral ethics. de los Reyes et al. (2017) have added more nuance to this assertion. They discuss the limitations of cognitive ethical models, like the virtues model, and the promise of newer models, such as behavioral ethics, that integrate much more realism about the human condition and argue for what they call a spaghetti model:

Ideally, the acceptance of a “spaghetti model” approach not only leads to blending conventional behavioral and philosophical insights, but also seeks to incorporate the kind of rich empirical and interpretive insights that are offered by various ethnographic and field study methods and from a wide range of social scientific theorizing. (de los Reyes et al., 2017, p. 332)

Our current research would fall within this spaghetti model. By focusing primarily on its cognitive dimension, ethics education has undermined the importance of social interactions in moral development. Although peer influence has been well researched in other contexts, it has not been systematically investigated within the teaching context of business ethics and corporate social responsibility courses.

Vygotsky’s sociocultural approach has been applied to other disciplines, such as cognitive development and moral development, but its application to business ethics has been virtually nonexistent (Kavathatzopoulos, 1993, 1994). This article contributes to the academic literature by advancing a Vygotskian approach to business ethics education, which, we believe, can offer a more robust account of how a peer/group dialogic process can have an influence on the moral reasoning of business students. Social interactions of peers in the learning process of moral reasoning will at the least allow for multiple perspectives to evaluate an ethical scenario and at best improve the moral reasoning of students in business ethics courses. It may even allow students to become business professionals with a strong sense of morality as they deal with complex ethical situations at the workplace. From a Vygotskian point of view, moral development is interdependent via social interaction with others, and these interactions can help students understand and create a personal meaning of morality.

In the next sections, we delve deeper into Vygotsky’s developmental theory and discuss its application to moral and business ethics education, followed by a discussion of peer influence in business ethics; these will lead to the presentation of the research questions. Thereafter, we continue to describe the data collection methodology, the type of data collected, and a discussion of the analysis and conclude with presenting some limitations, future directions, and practical implications. To start, let us outline the main tenets of Vygotsky’s developmental theory to explain how they might fit within business ethics education.

## Vygotsky’s Developmental Theory

Vygotsky’s developmental theory or theory of sociocultural development revolves around the following principles: children (learners) actively construct knowledge; learning is mediated by the use of tools and signs (language, symbols, etc.); language plays a critical and powerful role in shaping thoughts—that is, social speech (from social processes) becomes inner speech (psychological processes); and learning, which is socially constructed by inputs from others (peers, more knowledgeable others [MKOs]), and precedes development (Vygotsky, 1986; Vygotsky et al., 1978).

According to Vygotsky, the mechanism of cognitive developmental change is rooted in society and culture (Vygotsky et al., 1978, p. 7). Cognitive development is not a static process reached through the maturity of biological milestones; it is a dynamic process shaped and then reshaped by historical and material forces. He argued for a dialectical materialism where psychological processes are subject to change by looking at the relationship between thinking and language within social context. This means development is not linear but a dialectic process. As Vygotsky explains, psychological development does not happen in a circle but proceeds “in a spiral, passing through the same point at each new revolution while advancing to a higher level” (Vygotsky et al., 1978, p. 56). In this sense, higher cognitive functions originating from outside and being internalized is dialectic in the process, with language seen as the motivator of action both internally and externally. Burke (1966) has said that language is selective, abstract, and emotionally loaded and can create a diversion or separation. The diversion or separation can occur in the train of thought in the listener, particularly if the uttered words evoke responses other than what was intended by the speaker. Specifically, then, when a speaker uses language that evokes certain responses from the listeners (children, students, peers, etc.), depending on the context and the relationships between the speakers and listeners, the responses can become dialogic or deliberative. In the case of student-peers deliberating the ethicality of actions of protagonists in a business case, the language used by student-peers could elicit or evoke responses loaded with emotion and cause diversions or separations (particularly when there are multiple perspectives), and the iterative spiral of the dialogic or argumentation process can potentially lead to deeper insights.

For Vygotsky, the development processes involve the zone of proximal development (ZPD). The ZPD is based on the idea that learning should be matched to the child’s developmental level. For progression to occur, we must distinguish between a person’s actual level of development, which can be determined by previous tests, and their potential level of development if helped and prompted by a teacher or most knowledgeable other. The difference between the two levels is known as the ZPD. The ZPD defines problems that children (learners) cannot solve independently and gives them opportunities to cognitively develop with the help of assistance known as scaffolding. The ZPD also defines those functions that are not yet matured but are in the process of doing so. Most important, the ZPD gives educators and psychologists the space to understand how development occurs and to encourage development itself. It is the ZPD, we believe, which is important for business ethics education, coupled with learners being exposed to multiple perspectives to shape their critical reasoning abilities.

Much of the learning often takes place from knowledgeable peers or MKOs. MKOs often expose learners to new skills and capacities, which are then gradually internalized through direct teaching. Moreover, as the learner becomes better at those basic skills, they often turn to the MKO to deepen their understanding or knowledge or seek them out for further assistance, and thus the MKOs help (scaffold) learners to cross the ZPD. The acquisition of moral language can provide a good example of the relation between learning and development. For Vygotsky, “Language arises initially as a means of communication between the child and the people in his environment. Only subsequently, upon conversion to internal speech, does it come to organize the child’s thought, that is, become an internal mental function” (Vygotsky et al., 1978, p. 89). Vygotsky argues that the process of moral internalization, although complex, is founded on the relationship between the experienced MKO (peer or teacher) who helps the less advanced learner traverse the ZPD.

Although Vygotsky’s research focused on children, the ZPD has been applied to adults in several disciplines primarily through professional schools. For example, the ZPD was used in university education programs as a way of developing teaching best practices (Kuusisaari, 2014; Warford, 2011). In a recent study (Kantar et al., 2020), nursing students were paired with peers to help them with their clinical training. Peers encouraged students to reflect on their current skills and then reach beyond it using constructive feedback, prompts, and planned activities. In creating a ZPD, students were able to excel at treatments beyond their current skill level. Other applications of the ZPD include adults learning English as a second language both verbal and written (Aljaafreh & Lantolf, 1994). English as a second language students were paired with peers who provided language correction through a dialogic process. Collectively, both the student and the mentor co-constructed a ZPD, which allowed language learning to occur in a supported environment. In another study of Korean adults learning written English, the researchers found creating a ZPD, which provides careful corrective feedback to the learners, improves their knowledge of English composition compared with random correction and prompts by the MKO (Nassaji & Swain, 2000).

In a study spanning three semesters and eight courses on how adult university learners in face-to-face, blended, and online classrooms created and developed network relationships, Sundararajan (2010) found the emergence of peer MKOs to be an important factor affecting learners’ perception of conceptual and new knowledge gained. Using the ZPD as the basis of the emergence of the MKO, the author identifies that this approach allows the instructors and other peer MKOs to help lurkers and shirkers in the classroom be and stay more engaged with the course material. The formation of relationship networks among student learners during the semester had a positive impact on the motivation of learners to participate in class discussions, while interacting with their peers in the context of the course material.

By using the Vygotskian constructivist approach, Sundararajan et al. (2013) assessed the communication in student groups when they used face-to-face, instant messenger, and text messaging as key means of interacting with one another for subject matter–related discussions. The learners in the study were adults aged 18 to 30 years from first-year undergraduate to graduate students, a majority of whom had English as their first language, with about 22% indicating that their first language was other than English. The authors found that judicious and intermittent use of technology in the classroom helped mediate classroom discussions among student peers, and this increased their shared understanding of the subject matter and aided in learners gaining new and conceptual knowledge.

Vygotsky’s social constructivist approach to cognitive development can be bolstered by the work of Berger and Luckmann’s seminal work, *The Social Construction of Reality* (1966). Setting aside the philosophical questions of what or how we come to know the world, the authors take a sociological approach to knowledge itself. The sociology of knowledge suggests that individuals construct a meaningful worldview from interacting with their environment. Reality is seen as a construction from the words and deeds from others, which is then stitched together to form an objective whole with personal meaning. Human beings are knowledge carriers and interact as such through a dialectic process (Barns, 2016). Berger and Luckmann (1966) write, “To be in society is to participate in its dialectic . . .” (p. 149), which is then internalized, allowing us to understand each other and the world in a meaningful way. However, the authors, unlike Vygotsky, do add to an important emotional element to the concept of internalization. The dialectic process is not only part of a linguistic environment but is also often imbued with emotions and feelings. It is these emotionally charged elements of social constructivism that are necessary for the internalization and identification with specific ideas, concepts, and values.

Like Vygotsky, Berger and Luckmann (1966) argue language as essential to the society. Much of reality can be expressed by language—a sign that is the bedrock of any society. As the authors write, “Everyday life is, above all, life with and by means of the language I share with my fellowmen” (p. 51). Understanding language is essential to understanding reality and necessary to ensure a meaningful whole. However, unlike Vygotsky, language is also important for attaining different spheres of knowledge and their related semantic fields. Language creates meaning within “carved out” knowledge centers, which can then be taught and expressed linguistically. For example, plumbers, professors, poets, and politicians often have specific linguistic terms and references unique to their domain, which are usually only learned when we bump up against them (e.g., a clogged sink, attending university, etc.). Language defines not only what we know but also its boundaries. Knowing one is poor often includes restrictions on where one can live, choice of school, and career aspirations. In this sense, language plays an important role regarding the scope and limits of knowledge.

Although Vygotsky is unclear about the extent to which the dialectic process can affect cognitive development in adults, Berger and Luckmann (1966) leave open the possibility of such a change. They argue that the socialization process is never complete; it is ongoing. New ideas can shape and then reshape society again and again. In this sense, “Knowledge is a social product and knowledge is a factor in social change” (Berger & Luckmann, 1966, p. 104). Historical ideas and beliefs always stand in a dialectical relationship to those who challenge them. Hence, dialogue with peers suggests the possibility of new knowledge being constructed throughout one’s lifespan if challenged and confronted. We believe this dialogic process is important for changes in how people think about morality.

Although this literature review is not exhaustive, it should be clear that the ZPD applies to children and adults. Given the broad scope of the ZPD, we note that it can play a powerful role in improving moral reasoning of adult postsecondary students.

## Vygotsky and Moral Education

Vygotsky’s application of social constructivism to morality is limited. However, on the writings that exist, morality is equally mediated by language and discourse and thus subject to similar development processes. High mental processes, such as morality, move from the outer to the inner via social relations (Vygotsky, 1997). As Tappan (1991) explained, “The moral voices of justice and care enter the child’s psyche via different speaking voices that she hears in her socio-cultural world—in the context of her various social relationships and social interactions” (p. 249). Gradually, such external voices turn inward to become an inner form of ethical discourse.

Ethics is, broadly speaking, relative to society, culture, and class. Although Vygotsky is unclear to what extent morality is relative, language and social contents (contexts) shape moral thinking and actions. In this way, as a child (learner) progresses along their developmental path, an expectation is created by others that the learner will understand different points of view both cognitively (understand the feelings and emotions of others regarding a topic) and affectively (feel the same emotions as others regarding the topic). This is known as cognitive and affective empathy, respectively (Melchers et al., 2016). Empathy is an important psychological concept influencing social interaction. Studying 742 twins and nontwin siblings to investigate whether empathy is an inherited trait (nature) or a learned trait/behavior (nurture), Melchers et al. (2016) found that heritability estimates were between 52% and 57% for affective empathy, while it was much smaller at 27% for cognitive empathy. This indicates that some people are hardwired to feel others’ emotions, but for most people, it is a learned construct, specifically through and because of social interactions, both in their inner circle of family and outer circles of friends and acquaintances. Baker (2017) found that focusing on empathy helped students increase the awareness of self and others while prompting a more deliberate, thoughtful decision-making process when assessing ethical situations.

Although empathy is not discussed by Vygotsky, it falls within his social constructivist framework. Eisenberg and Mussen (1978), studying the relationship between empathy and two measures of moral development (prosocial moral reasoning and helping), found that mothers of highly empathetic boys were nonpunitive, nonrestrictive, egalitarian, and encouraged their offspring to discuss their problems and set high standards for their sons. Females’ empathy was not associated with parental socialization practices, and the researchers conjectured that this could be due to a ceiling effect. Hoffman (2000) similarly argued that empathy, which is essential to moral decisions, can be actively socialized in children through the mimicry of other behavior, conditioning, and the association between one’s response and situational context. Empathy can be learned through direct teaching from parents and modeling to cultivate prosocial attitudes leading to ethical actions. Additional research shows that children who are more empathetic also engaged in more care-based moral reasoning (Skoe, 2010), and empathy is associated with the quality of parent–child interactions. Children tend to show more empathetic prosocial behavior when raised in family settings where parents encouraged young children to recognize the needs and emotions of others and then engage in helping actions. The more parents talked to the child, the more oriented the child was to perspective taking (empathy) and acted in prosocial ways (Gross et al., 2015).

These findings are particularly relevant when we see that social interactions require peers to listen to one another actively and empathetically to understand what the other is saying and absorb that during the dialogic exchange. Such an exchange requires the processing of what they have just heard; responding in a manner that iteratively continues the discussion until the peers arrive at an understanding, a consensus, or an agreement; and then moving onto the next learning stage. Such a cross-pollination of perspectives lies at the root of social constructivism and social learning, leading to possibly deeper embeddedness of the concepts being debated or discussed.

Given the moral complexities in business, it also must be sensitive to culture and context and must recognize the importance of social peers for moral development. If correct, dialogue with peers can create a ZPD. We would like to offer some preliminary evidence to demonstrate that dialogue with peers can expose students to multiple perspectives and possibly even increase moral reasoning and development in students.

## Peer Influence in Business Ethics

A quick overview of Lawrence Kohlberg’s cognitive development theory (Kohlberg, 1984) is necessary because it is the foundation of our methodology and many other studies cited. First, moral reasoning can be defined as a process of “forming judgments about what one ought, morally, to do” (Richardson, 2013, para. 1). In other words, to engage in moral reasoning, a person needs to reflect on their values and order them in such a way as to justify specific moral decisions (Reimer et al., 1983, p. 45).

For Kohlberg, the moral maturation process involves moving through various stages and levels of development, which are invariant and universal, by starting at the preconvention level and then moving to conventional thinking about morality based on social conformity and obedience to authority. Although, according to Kohlberg, most people do not develop postconventional moral reasoning (the moral ideal), the last and the highest level is associated with human rights and universal ethical principles. For Kohlberg, exposure to divergent opinions in the social environment stimulates moral reasoning change.

Founded on Kohlberg’s theory, James Rest developed the Defining Issues Test or DIT2 (Rest, 1979) to assess increases or decreases in how moral actions are justified. The DIT2 has been widely used in the psychological and business ethics literature over the past 30 years and are widely recognized as suitable means for measuring moral reasoning (Thoma & Dong, 2014). Although we believe that using the DIT2 is an important metric for assessing moral reasoning change, we are not committed to the idea that rational reflection is the only means to moral development. As mentioned earlier, moral decision making is often influenced by external forces, such as situational specifics, reward systems, personality, and cognitive thinking, instead of merely evaluating whether actions are right or wrong (de los Reyes et al., 2017). Not recognizing the factors related to behavior does a disservice to ethics education. Schwartz (2017), using James Rest’s ethical decision-making process framework, provided pedagogical exercises and tools for teaching behavioral ethics that could help overcome certain barriers to ethical decision making, such as improper framing that can preclude moral awareness; cognitive biases and psychological tendencies that can hinder reaching proper moral judgments; and moral rationalizations that can obstruct moral judgments from being translated into moral intentions or ethical behavior. Such an approach allows us to also take the recommendations from de los Reyes et al. (2017) into our discussion. As we understand Vygotsky, understanding the role peers have on moral reasoning would fall within the scope of behavioral ethics and should be equally seen as influential.

The business ethics literature has few studies directly applying Vygotsky’s developmental theory to pedagogy. Only two studies are found, written by the same author, looking at the effects of instruction on subject’s ability to solve business ethics problems. In the first study, Kavathatzopoulos (1993) examined how instructions can promote autonomous decision making in adults. Participants were asked to solve five business ethics problems to assess their moral development and then instructed on ethical decision making. Finally, they were asked to solve the five business ethics dilemmas again. Results demonstrated instruction created a ZPD, which allowed participants to increase their capacity to solve moral dilemmas. Unfortunately, the author does not identify specifically how the ZPD was created and the specifics of the instruction are not clear. Given Vygotsky’s emphasis on peer discussion, details are essential to determine how the dialogue with the teacher took place, so this study does not truly reflect Vygotsky’s ideas.

Kavathatzopoulos (1994) rectified this problem in a follow-up study by adding group discussion into the methodology. Using 17 managers from a Swedish pharmaceutical company, each participant was given a pretest on ethics to determine their moral baseline and then lectured on business ethics. The managers were then broken into groups and were asked to solve moral dilemmas with the aid of the instructor. After discussion, groups presented their solutions for feedback to all the managers and finally completed a posttest. Results showed that instruction and group discussion had a positive impact on ethical problem solving. Unfortunately, Kavathatzopoulos does not sufficiently emphasize the importance of peer discussion or indicate how or in what way it was influential. Several nonbusiness studies have however demonstrated the importance of Vygotsky’s work related to moral development.

One of the first nonbusiness studies to specifically support Vygotskian theory comes from Turner and Chambers (2006). Based on the famous Heinz moral dilemma, the researchers asked students a series of questions to stimulate initial responses and then engaged the students in a group discussion. All participants in the study increased their moral reasoning following the group dialogue. The authors argue that moral learning primarily occurs in unstructured social environment rather than in “one off” formal courses or programs. In this sense, much of our moral learning seems to occur in social settings through an informal dialogic process, thereby supporting Vygotsky.

Other business ethics research, although not directly attributed to Vygotsky, have also emphasized dialogic processes to improve moral thinking. Dellaportas (2006) demonstrated improved moral reasoning in an accounting course through peer dialogue. Moreover, moral reasoning improvements using peer discussion have also been found in organizations, including managers (Trevino, 1986), auditors (Thorne & Hartwick, 2001), new employees (Keith et al., 2003), and salespersons (Pettijohn et al., 2011).

Discussing the pedagogical implications of delivering business ethics or corporate social responsibility courses to enable students to better address moral dilemmas in the workplace, Maclagan (2012) raises the option of using case studies, social interactions, and personal character to help resolve moral dilemmas. Aiming to investigate students’ understanding of business ethics issues on a sample of 307 management students at a Polish university, Tormo-Carbó et al., (2019) controlled for social desirability bias and found that perceptions of the course worsened after students had taken a business ethics course, female and older students displayed more ethical inclinations, and work experience was not a significant variable. The authors stress the need for further research in how business ethics courses are designed with a view to pedagogy, methodology of course delivery, and most important, the need to account for social, cultural, and contextual issues.

Innes (2006), while referring to problem-based learning student groups, stressed that communication within groups must be dialogic, and students must address problems using relevant concepts and deep principles and such an approach to construct useful knowledge that can be applied beyond the classroom. Kayes and Kayes (2003) found that the use of various pedagogies, such as lecture, simulations (Sundararajan, 2020) and experiential exercises, critical facilitation, and problem solving, can be used iteratively or conjunctively to facilitate deep learning. Ainsworth (2016) found that high-performing teams shared their research and knowledge with others, collaborated to advise and give constructive criticism, and demonstrated moral responsibility by respecting project management processes and communication protocols. Duarte (2009) advocated the adoption of C. Wright Mills’s sociological imagination in management education for its ability to foster reflection, critical thinking, and reflexivity skills.

Tomlin et al. (2017) found that blind spots create obstacles for ethics education and suggest that management education should include in-context ethics interventions that helps them recognize self-perception biases and that students should be trained to focus on self-reflection rather than broad normative principles. Hedberg (2017) avers that reflective learning practice be embedded across the business curriculum as a powerful way to equip students with intentionally formed moral habits of the mind and heart. While such an approach will be fraught with challenges, Hedberg provides educators ways to navigate the challenges and institute a moral reflective learning practice in business education. Eriksen et al. (2019) further support the use of self-reflection, practical reflexivity, peer coaching, and a class discussion of their experiences of attempting to live out their chosen virtues, what they are learning about being virtuous, and with what actions and structures they might experiment to improve their effectiveness.

Beggs et al. (2006) discuss the unique challenges in ethics education, the stark differences between what people feel as ethical/moral versus what is legal, and how we justify one or the other. They urge researchers and educators to continue to explore newer ways of engaging students with ethical concepts, dilemmas, and challenges to moral reasoning and that we all continue to espouse to our students the value of self-reflection in their learning behaviors and a commitment to leading ethical lives. In the next sections, we state our research questions, outline the methodology of our data collection process, present the results, and proceed to discuss them, all the while looking at how discussion among peers affects students moral reasoning and ethical/moral development.

## Research Questions

Given the above research, we argue business ethics education should be grounded in the ZPD with aid from the professor (the initial MKO in a classroom) and one who implicates reflective thinking based on dialogues between social and classroom peers. To explore this argument, we pose the following research questions:

**Research Question 1:** Does peer influence have an impact on students’ moral reasoning development and/or ethical decision-making abilities?
**Research Question 2:** Does receiving multiple perspectives on a workplace ethical dilemma have an impact on students’ moral reasoning development and/or ethical decision-making abilities?
**Research Question 3:** What is the effectiveness of having peer dialogue in business ethics courses?

Moral development is not restricted to childhood or adolescence; we have the capacity to improve our moral thinking and actions throughout our lifetime. Specifically, if moral functioning is an activity practiced in cultural contexts and mediated by words, language, and discourse, then a business ethics classroom and associated ethics training should equally create a ZPD for students that allows peer MKOs to emerge (Sundararajan, 2010) and help classroom learners traverse the ZPD. As we understand Vygotsky, morality is not indirectly discovered by introspective reasoning, but it is learned directly through peer discussion and reflecting on how to act and think. In the following sections, we will discuss our approach to answering the above research questions and lay out the foundations for the argument that business ethics classrooms can provide important social contexts to allow students to become better decision makers and businesspersons. To this effort, while we use dialogue and discussion interchangeably, in either case, for us to better explore the research questions, we would need the students to arrive at a decision when they are exploring the ethical cases as part of their team projects.

## Method

### Sample Description: Semester-Long Business Ethics Course

The study design involved teaching a semester-long business ethics elective to undergraduate students at a Western Canadian university. The classes predominantly comprise business majors, with a few students studying other disciplines (psychology, justice studies, and journalism). In all, 29 students consented to participate.

### Sample Description: Management Skills Development Course

To reinforce our overall thesis, we compared the business ethics data with a semester-long management skills development course from an Eastern Canadian university. We wanted to compare the semester-long course with a management class that had little ethical content and peer discussion about moral issues. A course devoted to business ethics, we argued, should have greater effect at increasing the moral reasoning of participants compared with a nonethics focused management course. The objective of the management skills development course was to expose students to key knowledge, skills, and competencies to ensure management success. Its applied nature focused on effective management practice, including a short section on ethical behavior.

### Data Collection

The business ethics course was the experimental group, and participants completed several assessments and assignments:

1. At the beginning of the semester, participants took the DIT2 before any ethical content was taught to establish a baseline of moral reasoning (Rest, 1979). The course then proceeded in traditional fashion, including an overview of ethical theory and then an investigation of specific moral issues, such as CEO pay, whistle-blowing, insider trading, and bribery, to name a few. Each topic was assessed regarding the application of moral theories and an analysis of specific arguments for and against. Participants were then asked to complete the DIT2 postcourse to determine whether there was an increase in moral reasoning.

Traditionally, the DIT2 assessed the level of moral reasoning by the P score, which ranked the individual’s response to cases based on postconventional thinking. However, the P score only looks at postconventional reasoning, and lower states of moral development are dismissed. To correct this problem, the N2 score was devised to take into consideration P scores and an individual’s ability to discriminate between other stages (Thoma, 2006). Collectively, the N2 score provides a more accurate representation, and therefore, we used N2 scores in this study to determine levels of moral reasoning in our participants.

2. To facilitate ethical thinking, case studies were used throughout the term. Participants were divided into groups of three to five people and asked to read, discuss, and then solve five business ethics case studies (whistle-blowing, discrimination, outsourcing/sweatshops, marketing, and environmental) over the semester. There was no student training regarding how students engaged in group discussion. The semistructured questions were sufficient to stir student conversation. During the discussion period, participants were asked to respond to questions about the moral dilemmas. The questions were open-ended, which allowed flexible group discussion, and all members were encouraged to participate and express their thinking and resolution to the moral problems. Deadlines were established for submission of group responses to the ethical dilemmas. Of each of the 24 eighty-minute classes over the semester, for approximately 30 to 40 minutes each week, students actively engaged in group discussion about specific cases and then drew conclusions, which were then posted on an electronic discussion board that allowed researchers to retrieve them to conduct content analysis.
3. To assess the effects of such group dynamics on discussion, participants were asked to reflect on how and to what extent peers influenced their ethical decision making by answering a series of open-ended questions, which were then posted in an electronic journal. The main categories we focused on were consensus, effects on shy students, understanding ethical concepts and knowledge, equal contribution, and peer discussion impact on participant’s ethical decision making. More specifically, we were interested in participant responses to the question: Did group-peer discussion influence your ethical decision making? If so, how and in what way? If not, why not?

As for the management skills development course, adding a degree of complexity to the study, the participants were divided into experimental and control groups. The experimental group consisted of the following: (1) participants were once again asked to complete the DIT2 before being taught ethical theory basics, which took about a week; (2) groups then responded to a business ethics case through discussion. Groups were self-selected at the beginning of the semester and had one week to respond to the case questions. (3) Finally, students were asked to complete the DIT2 postclass followed by a series of questions on peer influence, identical to our full-semester business ethics experimental group.

For the control group, participants were also taught ethical theory and then individually responded to a case on outsourcing/sweatshops by answering a series of questions following the same timeline as above. Students did not discuss the case with peers but merely submitted their answers to an electronic journal, which will not be discussed here. There were no individual journals submitted because no peer discussion took place. In all cases, approval was received from the research ethics boards of both universities.

### Data Analysis

The DIT2 test results were analyzed based on average N2 scores for both the business ethics and management courses. The N2 score measures the extent to which participants’ prioritized statements associated with Kohlberg’s postconventional thinking and can have a range from 0 to 95. An increase in N2 score for participants shows progression toward higher levels of postconventional moral development. Average N2 scores were compared against the experimental and control groups and between men and women.

Since participants were tasked with answering the question about peer influence, content was analyzed using QDAMiner with Wordstat. QDAMiner has a text mining feature that allowed us to systematically code all group journals and then create frequency percentages. Among the various reasons why content analysis is used, some are the need to ascertain the intent of communication trends between individuals or groups (or greater agglomeration of entities—departments, etc.), the nature of interactions between individuals or groups, responses on open-ended questions from surveys or focus groups, and possibly even attitudinal or behavioral patterns emerging from communication between individuals and groups. Typical approaches to content analysis have taken either a conceptual (existence or frequency of concepts in a text) or a relational (where relations exist among the concepts in the text) method. Since we were looking to identify the presence of elements of argumentation and decision making as well as the presence of conceptual ethical frameworks in student discussions around course concepts relating to moral reasoning, we adopted the conceptual approach to content analysis. This allowed us to provide an objective, systematic, quantitative description of the manifest content (Berelson, 1952) in student responses and discussions.

**Figure 1.**
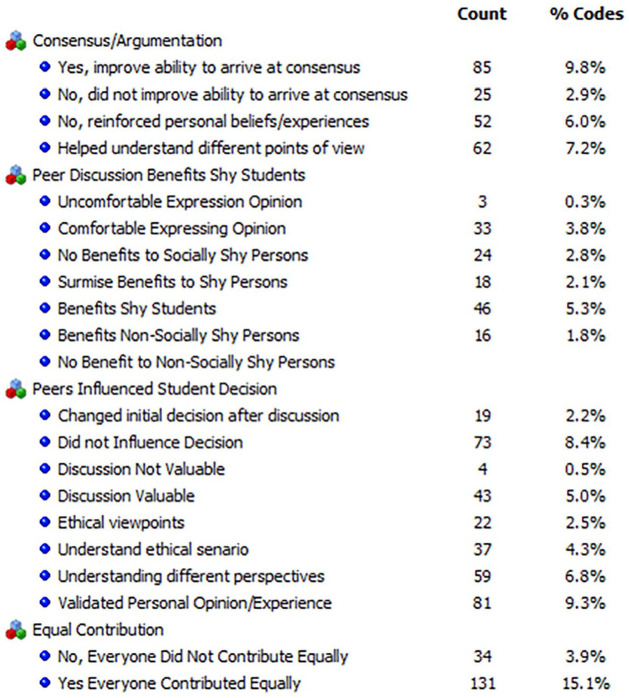
Code frequencies—student journal entries—Western Canadian university.

**Figure 2.**
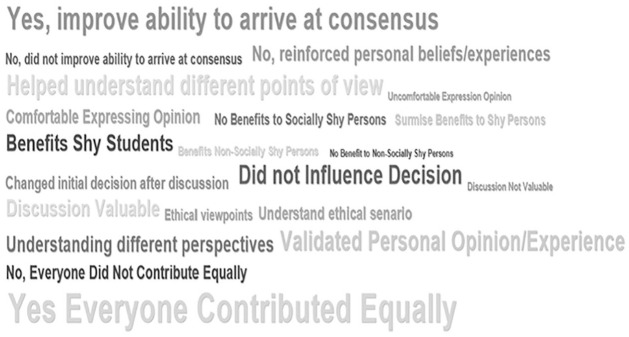
Code frequencies—student journal entries—Eastern Canadian university.

Taking a thematic coding approach (Krippendorff, 2004), we adopted the codes around argumentation developed by Sundararajan et al. (2013) to ascertain whether the discussions led to consensus or agreement. Prior to collecting data from the archived individual journal entries, two of the researchers in the team set about identifying the definitive themes for the code categories. Student journals were copied into a word document and then uploaded into the QDAMiner software system. Since we have used and adapted the codes (the adapted thematic code categories are illustrated in Figures 1 and 2) created by Sundararajan et al. (2013), we used the deductive approach to coding the text.

The lead author and the second author performed the initial coding on the data sets. For this, we took a subset (identical samples) from the data set and coded them independently based on the thematic code categories that we adapted. The unit of analysis was at the sentence and word levels, where we needed to check whether words that indicated “I agree” and “I disagree” around the argumentation codes and “I changed my ethical views” and “I did not change my ethical views” around the peer discussion codes. QDAMiner allows each coder to create a separate account on the data set, and when the independent coding was completed (on the subset of the data set), we ran the intercoder reliability procedure using the Krippendorff Kappa statistic. After the initial coding effort, we had an intercoder reliability of about 60% to 65%. A typical acceptance reliability rate is above 75%. We discussed aspects of the codes and resolved the differences on how we would code the data segments. The resolution of differences centered on how we interpreted what participants meant when they responded to the questions on case-related ethical issues, agreements around discussion points with their peers, and benefits of discussion. Additionally, we added thematic codes around the viability and usefulness of discussions to facilitate learning, collaboration, changing of one’s initial decision on the ethical/moral views on the case, overcoming social barriers (shyness, etc.), and whether everyone contributed equally to the discussions. This resulted in a near perfect agreement on what each code meant. These codes are presented in Figures 1 and 2.

**Figure 3.**
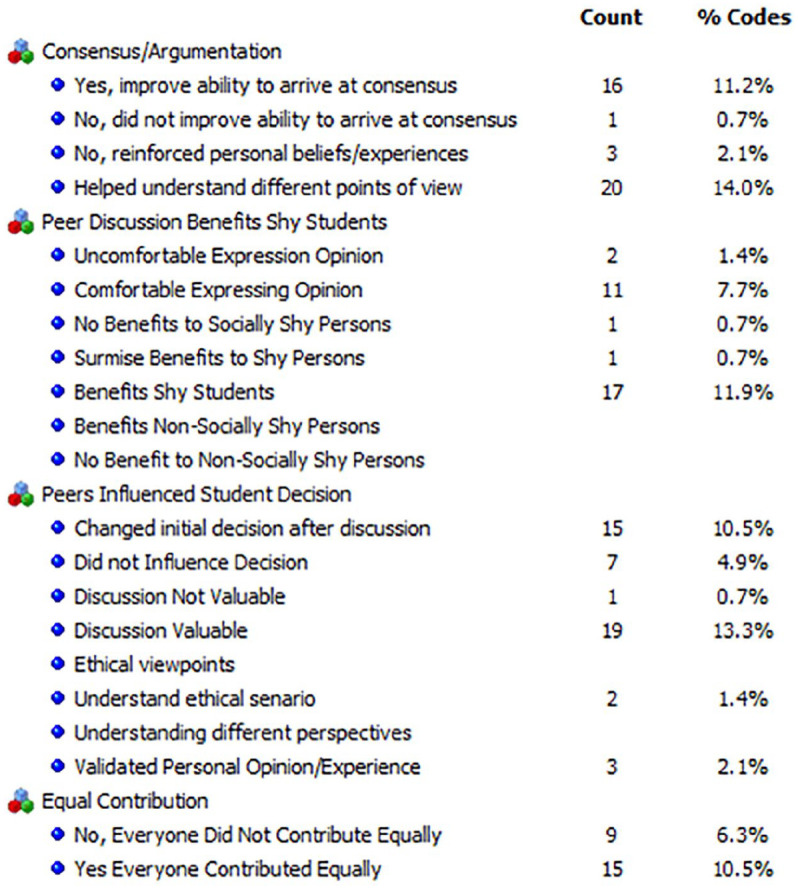
Word cloud code frequencies—student journal entries—Western Canadian university.

**Figure 4.**
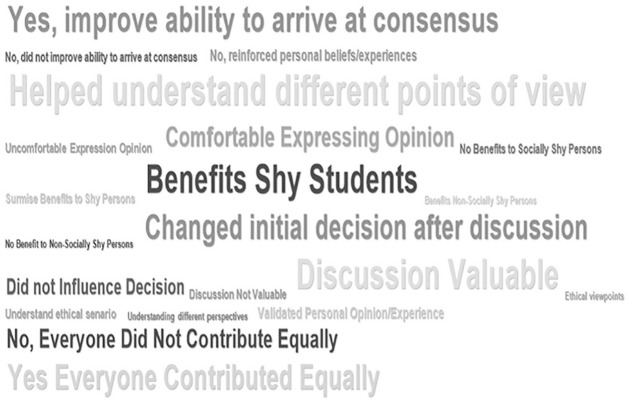
Word cloud code frequencies—student journal entries—Eastern Canadian university.

Student responses were straightforward and direct with little need for interpretation. For example, students often stated there was “no influence,” “my beliefs were unchanged,” “unwilling to budge on my personal views,” “didn’t influence my ethical reasoning,” or “my ethical decision was not changed,” to provide a few examples. Once coded, a frequency calculation relative to the number of journals was completed, allowing us to depict the word clouds for the frequency of occurrence of these thematic codes in the students’ responses (Figures 3 and 4).

## Results

### Defining Issues Test

For the experimental business ethics course, only 29 students agreed to participate, but on an average, they increased their moral reasoning level from 36.03 to 43.21. Of those DIT2 postsurveys, 20 (69%) participants increased their moral reasoning, while 9 (31%) participants decreased their moral reasoning (Table 1). While both men and women participants increased their moral reasoning, there was no significant difference between them. Interestingly, this contradicts previous research that suggests that women score higher than men (Thoma, 1986).

**Table 1.** DIT2 Business Ethics Course on N2 Scores (Western Canadian University).

Demographics	DIT2 N2 score precourse (mean)	DIT2 N2 score postcourse (mean)	Difference	Percentage change
Total (*N* = 29)	36.03	43.21	7.17	19.92
Increased moral reasoning (*N* = 20)	31.29	45.33	14.03	44.87
Decreased moral reasoning (*N* = 9)	46.57	33.94	−12.62	−27.12
Female (*N* = 12)	31.98	42.68	10.70	33.45
Male (*N* = 17)	41.77	43.95	2.17	5.21
Diverse groups (*N* = 13)	38.90	42.85	3.9	10.15
Nondiverse groups (*N* = 16)	33.71	40.94	7.22	21.44

*Note*. DIT2 = Defining Issues Test.

As for the management skills development course (Eastern Canadian University), although 42 students agreed to participate, only 24 had usable data. Participants were evenly distributed between experimental and control groups. The DIT2 pre- and postdata reveal the experimental group’s (*N* = 12) moral reasoning decreased modestly from 39.83 to 37.44, while the control group (*N* = 12) saw a more significant overall decrease from 40.21 to 32.31 (Table 2). Although both men and women had overall decreases in moral reasoning, the control group experienced much greater overall losses. The same cases were used for both experimental and control groups.

**Table 2.** DIT2 Management Skills Development Course N2 Scores (Eastern Canadian University).

Demographics	DIT2 N2 score precourse (mean)	DIT2 N2 score postcourse (mean)	Difference	Percentage change
Total control no peer discussion (*N* = 12)	40.21	32.31	−7.9	−19.64
Increased moral reasoning (*N* = 4)	25.97	40.28	14.31	55.10
Decreased moral reasoning (*N* = 8)	47.33	28.33	−19	−40.14
Female (*N* = 8)	42.13	38.46	−3.67	−8.71
Male (*N* = 4)	36.37	20.01	−16.36	−44.98
Total experimental peer discussion (*N* = 12)	39.83	37.44	−2.39	−6.00
Increased moral reasoning (*N* = 5)	29.45	39.36	9.91	33.65
Decreased moral reasoning (*N* = 7)	47.25	36.25	−11.18	−23.28
Female (*N* = 9)	38.26	35.77	−2.49	−6.50
Male (*N* = 3)	44.54	42.44	−2.10	−4.71

*Note*. DIT2 = Defining Issues Test.

### Individual Journals

In total, there were 208 journal entries by 29 participants in the business ethics experimental group. When answering the question about whether group-peer discussion influenced one’s ethical decision making, participants provided a variety of unprompted responses, but only 4.3% participants indicated that peers had such an impact on understanding the ethical scenarios and ethical viewpoints (2.3%). Despite some participants changing their opinions (2.2%), 8.4% indicated peers had no influence on their ethical decisions, while another 6.4% said discussion merely reinforced personal opinions. So discussion and debate (argumentation) within groups helped some students craft arguments to validate and justify their own opinions. Participants stated that groups were crucial for helping them understand different perspectives or the ethical case study itself (7.2%) and improved the ability to arrive at consensus (9.8%). Participants also felt that discussion benefited shy students (5.3%) and surmised that it might have benefited shy students (2.1%). Overall, only 0.5% of participants said group discussion was not valuable. A word cloud of the codes indicates a similar picture (Figure 3). Word clouds are simple representations of the frequencies of the codes that are being studied/analyzed. The QDAMiner application provides options to depict frequencies in terms of bar/column graphs, pie charts, or word clouds. The higher the frequency of a particular word or thematic code category, the larger the font size in a word cloud and brighter the color of the code being depicted. For this reason, we chose to depict the thematic code frequency (occurrence of the code) in the form of a word cloud, as opposed to any other type of chart.

As a next step, we conducted code sequence analysis. In this procedure, we looked at what coded segments occur next to one another and how frequently does this happen in the analyzed text. The QDAMiner application allows us to select codes of interest, such as a coded segment indicating collaboration and a coded segment indicating benefits of discussion or a coded segment on the usefulness of discussion and ability to appreciate different viewpoints or perspectives, and compare these codes against one another to (1) determine which of these code segment pairs co-occur (in sequence) and (2) determine whether the frequencies of these co-occurrences are statistically significant using *Z* scores. *Z* scores or standard scores allow us to calculate/measure how far the standard deviations of the raw scores are above or below the population mean. Additionally, this approach standardizes the raw scores into a normal distribution. When we look at how the code sequences stack up in a code sequence analysis (Table 3), “Yes, Everyone Contributed Equally” is significant with “Changed Initial Decision After Discussion” (*z* = 2.48, *p* = .023), “Comfortable Expressing Opinion” (*z* = 1.69, *p* = .056), “Discussion Valuable” (*z* = 3.04, *p* = .005), “Helps Understand Different Points of View” (*z* = 3.27, *p* = 0), “Understanding Different Perspectives” (*z* = 2.02, *p* = .038), and “Validated Personal Opinion/Experience” (*z* = 2.05, *p* = .033).

**Table 3.** Code Sequence Analysis—Significant Results—Western Canadian University.

Code A	Code B	Frequency A	Frequency B	Frequency (B|A)	Frequency (A|B)	z	*p*
Yes, everyone contributed equally	Changed initial decision after discussion**	131	19	8	8	2.48	0.023
Yes, everyone contributed equally	Comfortable expressing opinion*	131	33	2	2	−1.69	0.056
Yes, everyone contributed equally	Discussion valuable**	131	43	16	16	3.04	0.005
Yes, everyone contributed equally	Helped understand different points of view***	131	62	1	1	−3.27	0
Yes, everyone contributed equally	Understanding different perspectives**	131	59	17	17	2.02	0.038
Yes, everyone contributed equally	Validated personal opinion/experience**	131	81	22	22	2.05	0.033

* *p* ≤ .1. ^**^p < .05. ^***^p < .001.

When code sequence analysis is performed, it is also good practice to look at the search hits or the codes in context to ascertain whether what is being said has been coded correctly. In the interest of space, we present in Table 4 a few snippets of “Everyone Contributed Equally” versus “Discussion Was Valuable.”

**Table 4.** Code Sequence Analysis Sample Code-in-Context Search Hits—Western Canadian University.

Case	Yes, everyone contributed equally	Discussion valuable
Phil 2229 individual journal responses	Everyone contributed equally to the decision-making process when we were discussing the ethical issues.	It was a very good group and we were all feeding off each other’s thoughts and contributing to the discussion
Phil 2229 individual journal responses	Everyone equally contributed to the final decision	It made me feel like that my thoughts are also being valued and taken into consideration, and I’m sure everyone else who usually don’t get the chance to express their feelings and ideas got the opportunity to express their ideas.
Phil 2229 002 individual journals	Contributed equally and make sure that everyone’s opinion is accounted for. We listen to what each person has to say and then decide what the best approach for	Thinking when a person can thoroughly back up their opinion and demonstrate to me why their approach or view is more appropriate than mine for any given case.

For the management course, there were 35 journals from the experimental group, with 11.2% participants indicating that the argumentation improved their ability to arrive at consensus, while 14% indicating that it helped understand different viewpoints. Another 7.7% indicated that they were comfortable expressing their opinions, and 11.9% indicated that it benefited shy students. Regarding peers influencing their decisions, 10.5% indicated that they changed their initial decision on the ethical scenario after the discussion, while 4.9% indicated that the peer discussion did not influence their initial decision. However, 13.3% indicated that the discussion was valuable. Figure 2 (code frequencies chart) and Figure 4 (word cloud) illustrate these results.

Looking at the code sequence analysis (Table 5), we noted that when “Everyone Contributed Equally,” participants reported that they “Changed Initial Decision After Discussion” (*z* = 4.05, *p* < .001) and participants found the “Discussion Valuable” (*z* = 1.99, *p* = .0056). Table 6 presents some of the code-in-context hits.

**Table 5.** Code Sequence Analysis—Significant Results—Eastern Canadian University.

Code A	Code B	Frequency A	Frequency B	Frequency (B|A)	Frequency (A|B)	z	*p*
Yes, everyone contributed equally	Changed initial decision after discussion***	15	15	8	8	4.05	0.001
Yes, everyone contributed equally	Discussion valuable*	15	19	6	6	1.99	0.056

* *p* ≤ .1. ^**^p < .05. ^***^p < .001.

**Table 6.** Code Sequence Analysis Sample Code-in-Context Search Hits—Western Canadian University.

Case	Yes, everyone contributed equally	Changed initial decision after discussion
Individual business ethics response Eastern University experimental	Voice our opinions freely	Influenced my ethical decision making
Individual business ethics response Eastern University experimental	Everyone was equally contributing to everything	Definitely had an influence on my ethical decision-making
Individual business ethics response Eastern University experimental	As far as I can recall, everyone participated equally	Did, mainly because I saw other points of view from my own. I’m very set in my ways so it was refreshing to get a different perspective. The situation wasn’t so black and white.
Individual business ethics response Eastern University experimental	Yes. It makes me feel that this is a really perfect cooperative group. Everyone is working hard and working hard for the team. It makes me feel a sense of belonging	Convince other members. But also the other team members can convince me. When our point of view conflicts, we will argue and persuade to get an agreement.

### Note on Quantitative Analysis of the DIT2 Scores

For the 65 participants in the semester-long business ethics courses, we only obtained DIT2 scores for 29 participants. While the DIT2 scoring performed by the University of Alabama only provided the DIT2 N2 scores and the mean differences (pre- and postcourse), we performed a paired sample *t* test on these N2 scores. The mean precourse N2 score was 35.354 and the mean postcourse N2 score was 41.363. For this paired sample *t* test, *t* (critical-28) was 2.052 (*p* = .014), indicating that the increase in the N2 scores postcourse was statistically significant when compared with the N2 scores precourse.

For the management skills development course, we were able to obtain only 16 completed DIT2 surveys (pre- and postcourse) for each of the control and experimental groups. However, much of the data had to be purged as many participants did not complete the surveys and we had only 12 useful surveys. The mean precourse N2 score for the control group (where there was no group discussions) was 39.85, and the mean postcourse N2 score was 41.58. For the control group, the *t* (critical-12) was 1.753 and *p* was .78 and hence not statistically significant. For the experimental group, the mean precourse N2 score was 29.603, and the mean postcourse N2 score was 44.510. For this group, the *t* (critical-12) was 1.75 and *p* was <.001 and hence statistically significant.

For both the semester-long business ethics course and the management skills development course, while we performed *t* tests on the DIT2 N2 scores and found statistical significance between the pre- and postcourse N2 scores, we refrain from discussing this in detail as in addition to the sample sizes being small, we were not performing hypothesis testing at this stage of the study.

## Discussion

Our study shows that peer discussion plays an important role on moral reasoning and supports the premise that creating a classroom or ZPD where students feel safe to express and exchange opinions with others about moral issues is necessary for development. Following Vygotsky, social interaction is crucial to improve moral reasoning. The semester-long course, unlike the shorter inclusion of ethical content in the management course, allowed participants to continually discuss and thus increase their competence in understanding and applying ethical concepts and theories. It is this kind of development that clearly has a much greater impact on moral reasoning than one-off learning.

The business ethics course, unlike the management skills course, offered participants a more immersive experience in ethical “culture” and greater learning from MKOs—instructor and peers—over a longer period. In the business ethics course, ethical concepts and theories were continually applied throughout the semester and many participants became competent in their application. Not only were the MKOs important for teaching ethics, but as participants sought clarification or desired to strengthen their knowledge, discussions allowed them to engage with MKOs in dialectic or spiral fashion. However, this learning can only take place if there is sufficient time to deepen ethical understanding. Compared with the business ethics class, a smattering of ethics education into management curriculum is insufficient to significantly improve moral reasoning.

In the control group, which had no peer discussion, moral reasoning dropped precipitously and underscored the importance of peer discussion on moral reasoning. Despite some modest ethics training, participants who did not engage in peer discussion saw an overall decrease in their moral reasoning levels. It appears that ethics education, including the use of MKOs and peer discussion, is necessary to achieve significant moral reasoning gains.

Each of these results viewed in the context of the limited sample size give us the indication that we should continue to explore this approach for creating student and peer discussions, where everyone in the group is contributing equally and then are able to realize the benefits of the peer group learning process. More data will be necessary to draw firm conclusions, but prima facie journal responses suggest peer discussion had some influence on the moral decisions of participants, where some changed their initial decisions, others indicated that their decisions were validated by the discussion/debate, and discussions allowed shy participants to contribute and benefit. Most important, peers influenced how students reasoned about moral problems, as evident in increases in the DIT2 results (answering Research Question1). Together, these results indicate that peer deliberation/discussion does provide students with multiple perspectives on ethical scenarios and related decision making (answering Research Questions 2 and 3). If Vygotsky is correct, learners continually revisit and recast ideas and concepts as they grow and develop a more robust understanding of them. This means the instructor or MKO needs to scaffold student learning needs more, and in doing so, the concepts become internalized to the point where they are mastered. However, given the limited instruction on ethics in the management course, we suggest that for some students, the ethical concepts are not mastered or internalized but merely mimicked back to the MKOs; hence, the higher levels of peer influence are noted. As moral concepts become internalized, they get integrated, assessed, and analyzed through the individual’s experiences, historical knowledge, education, and emotions. Concepts get contrasted with an individual’s own thinking about ethics and how they might affect their lives, if at all. However, such internalization takes time, and most important, if new moral concepts are not mastered or if ethical issues are not seen as problematic and needing to be solved, “thinking fails to reach the highest stages, or reaches them with great delay” (Vygotsky, 1986, p. 108). We are arguing that the limited business ethics education and peer discussion within the management course insufficiently created the motivation to solve such ethical problems and thus is not truly internalized by participants. Moreover, the DIT2 results show that the management class had higher levels of conventional thinking than the business ethics course, which places greater emphasis on social conformity (i.e., participants might feel embarrassed to disagree) and thus a greater percentage of participants should express more belief change. Conversely, for the business ethics class, higher levels of postconventional thinking would result in more independent moral thought irrespective of group dialogue or peer pressure.

The data in this explorative foray clearly fall into Vygotsky’s idea of social constructivism. The DIT2 tests demonstrate that group discussion helped some students achieve their potential development through the internalization of alternative perspectives. Moreover, the reflective journaling allowed the students to write down their thoughts on the ethical dilemma faced by the actors in the case, and the peer dialogue that followed enabled them to either validate their own individual responses (as it appears in most of the data) or change their decisions (as it appears in some of the data).

But caution should be exercised regarding to what extent peers will change or alter the beliefs or values of their colleagues. Social psychologies have raised considerable doubt on the effectiveness of peer discussion on moral belief change. Haidt (2001), for example, has argued that it is intuition, not rationality, which is the main building block of moral decision making. Moral decisions are, really, just flashes of what we think are good and bad with little, if any, rational deliberation at work. Moral reasons to support a decision are provided post hoc to justify and support preconceived beliefs. This means the arguments and reason presented to others have little influence on other’s thinking about moral problems. The conclusion is supported by our evidence. Very few students in the business ethics and management skills development classes claimed their moral beliefs changed because of peer discussion. However, this does not mean peer discussion is not valuable, as we have seen from the analysis of the small data set. As stated previously, most students in the business ethics course increased their level of moral reasoning from preconventional/conventional to postconventional thinking. In other words, despite minimal change to moral beliefs, there is considerable change in how these beliefs are justified and reasoned.

### Limitations and Future Research

The small sample sizes in both experimental and control groups are an obvious limitation. However, we still had 29 useful responses with 208 journal entries from the Western Canadian University and 24 useful responses with 35 journal entries from the Eastern Canadian University to analyze. We will need a much larger sample size before definitive conclusions can be drawn. However, the trends in the data should not be dismissed. A Vygotskian approach to moral education, as demonstrated in our results, is encouraging and provides an exciting opportunity for researchers to explore more fully.

As mentioned earlier, there are important conceptual differences between dialogue and discussion. Although groups tended to engage in discussion, as opposed to dialogue, its inclusion in future research might affect our results. For example, Graybill and Easton (2015) point out that dialogue does not happen naturally. Often, instructors must take a more Socratic approach when it comes to stimulating dialogue. Providing students with open-ended questions or using phrases such as, “perhaps, I wonder,” and “what if?” are two ways of getting the listener to reflect on what the topic, idea, or concept means to them (Graybill & Easton, 2015). The individual meaning can then be expressed back to others in a respectful environment where perspectives can be shared to achieve collective understanding. However, group understanding is premised on, as Graybill and Easton suggest, slowing the tempo, allowing each person uninterrupted time to talk, and listening carefully to each other. Dialogue, in this sense, can be used as a precursor to discussion.

We will next discuss the practical applications to this approach and how instructors teaching business ethics can better engage their students with the ethical concepts and look to having the students start the process of internalizing these concepts as they navigate their education and in their workplace.

### Practical Applications and Concluding Thoughts

The research presented has several practical implications for business ethics instruction. First, case studies have been widely used to teach business ethics for decades and are considered an effective method compared with traditional lecture-style teaching (Waples et al., 2009). The importance of case-based instruction has been expanded to a host of other disciplines, such as chemistry, nursing, and biology (Bonney, 2015). Research has shown that case-based instruction promotes analytical thinking and concept understanding, helps identify specific learning objectives, provides real-world application, and increases student motivation to participate. However, we argue that case studies must be coupled with group discussion to be truly effective. Instructors should pick cases carefully to ensure they are relevant to the objectives of the class. Case studies should be open ended with no clear answers for students. We believe that it is best to find cases that are “backward looking.” Meaning the ethical issue has been solved, and there is enough information to allow the instructor, once discussion of the case has occurred, to “reveal” the case conclusion. This gives students a way to compare their own ethical decision making and actual managerial thinking.

Second, although our results provide evidence that small group discussion improves the overall moral reasoning of students, the data also reveal that diverse peer discussion might be even more important than peer discussion itself (see Table 1). For the business ethics course, some participants selected their own groups and remained in them throughout the semester. Personal observation and individual journal feedback from participants reveal many students chose colleagues who were in the same disciplinary study, ethnic background, or gender. At other times, the professor changed groups with each case to ensure greater diversity within group discussions and decisions. Diversity, in our case, includes age, gender, ethnicity, conservative or liberal, and religious orthodoxy. This is consistent with other research (Gurin et al., 2002; Hurtado et al., 2012), which shows being exposed to peers with diverse racial backgrounds, political views, religious beliefs, and ethnic histories can expose students to important alternative perspectives.

In forming groups, instructors should do so with forethought. Diversity in groups is important because it offers students an opportunity to listen to voices from different points of view and perspectives. To ensure diversity, one of the authors handed out a short “demographic” form to students prior to assigning groups. The form consisted of basic information, such as gender, year of study, major, travel experience, whether they spoke a second language, and future occupation. In the diverse-groups condition, this information was used to form groups to create as much diversity as possible. A Microsoft Excel spreadsheet was used to track students as they switched to new groups for every case discussed over the term.

Third, instructors are encouraged to create a ZPD so students can comfortably discuss moral issues with others as a way of thinking about their own personal values and beliefs. At the beginning of the term, for example, icebreaker exercises in student pairs are a great way for them to meet each other. As groups are formed, students will naturally get to know their colleagues. The more comfortable students are with each other, the more they will share their beliefs and decisions about the moral cases discussed in class. Once in groups, students will also quickly discover which of them best understands the concepts, theories, and ideas presented in class. These MKOs are important for encouraging other students to learn from them and have students observe and practice their skill at applying ideas and concepts to the cases. Instructors also play an important role in scaffolding or providing supporting activities for students through lectures and general understanding of ethics. More specifically, as instructors, it is imperative to cycle through the classroom when groups are discussing cases and take time to ask questions about the nature of the case, key concepts, and potential solutions. In the classroom, while professors are the ultimate MKO, such an approach allows for the emergence of peer MKOs (Sundararajan, 2010).

Finally, engaging in group discussion and allowing students to think deeply about moral problems is organized chaos, but it is rewarding. Philosophy, including ethics, is not mere navel gazing; it is something you must do. It must be talked about, discussed with others, and debated to understand its concepts, theories, and how they can be applied to real-life situations. However, this means that before classes start, instructors must develop the course outline in such a way as to enable peer discussion to take place while meeting course content demands and objectives. Monitoring is also important to ensure student stay “on task.” But the rewards are worth it; students take their group decisions seriously and tend to work through the problems with diligence and efficiency.

## Conclusion

The application of Vygotsky’s development theory can provide important insights for business ethics education. Waples et al. (2009) suggest shorter durations (no more than 30 days) to be most effective, although they do not directly track peer discussion as an important influencer on ethical education; they do, however, state that students should be “heavily engaged in the learning process” (p. 147). We argue that peer discussion within a ZPD should be part of this heavy engagement of which Waples et al. speak. Second, we believe there is value in students discussing and debating moral problems as a way of informing and refining decision-making strategies. Finally, because Vygotsky argues development is a continuous process, not staged, learning should be a collaborative and continuous process. Also, student beliefs and values must be openly and safely challenged through social interaction as a way of encouraging reflective thinking about business ethics. Instructors of business ethics should create a ZPD so peers can help and assist others to think and talk about moral issues in new ways and recalibrate their moral thinking (Ohreen, 2013). Peer dialogue within a business ethics course can spur students to challenge their own assumptions and beliefs, thereby enhancing their conscious moral thinking. However, such changes cannot occur until we recognize moral development, not as an isolated affair, but as a series of “concrete interactions between . . . adults talking and acting together in groups” (Miller, 2011, p. 32). Business ethics education should be part of a student’s broader moral development.
